# Genetic prediction of the casual relationship between micronutrients and ER+ breast cancer: a Mendelian randomized study

**DOI:** 10.3389/fgene.2025.1599724

**Published:** 2025-07-23

**Authors:** Wei-Da Fu, Jin-Qiu Wang, Jin Luo, Ming-Ze Wei, Yong-Ping Dai, He-He Wang

**Affiliations:** ^1^Department of Thyroid and Breast Surgery, The First Affiliated Hospital of Ningbo University, Ningbo, Zhejiang, China; ^2^Department of Otolaryngology, Head and Neck Surgery, The First Affiliated Hospital of Ningbo University, Ningbo, Zhejiang, China

**Keywords:** micronutrient, estrogen receptor-positive breast cancer, vitamin B6, Mendelian randomization, risk factor

## Abstract

**Background:**

Estrogen receptor-positive (ER+) breast cancer, a prevalent subtype of breast malignancy, demonstrates complex etiological associations with multiple risk factors. Micronutrients, as essential nutritional components for human physiology, may potentially influence the pathogenesis and progression of breast carcinoma. This investigation employs Mendelian randomization (MR) methodology to assess causal relationships between 15 micronutrients and ER+ breast cancer.

**Methods:**

In this study, instrumental variables (IVs) for 15 micronutrients were extracted from the genome-wide association studies (GWAS) database, including copper, calcium, carotene, folate, iron, magnesium, potassium, selenium, vitamin A, vitamin B12, vitamin B6, vitamin C, vitamin D, vitamin E, and zinc. Concurrently, summary data related to ER+ breast cancer were obtained from the FinnGen database. Following the selection of appropriate IVs, we conducted a two-sample MR analysis. This analytical framework incorporated comprehensive sensitivity analyses to evaluate potential heterogeneity and horizontal pleiotropy, with the inverse variance weighted (IVW) method established as the principal analytical approach.

**Results:**

The findings of our study revealed a significant causal relationship between vitamin B6 and ER+ breast cancer. Notably, genetically predicted elevated vitamin B6 levels were significantly associated with an increased risk of ER+ breast cancer [Odds Ratio (OR): 1.275; 95%Confidence Interval (CI): (1.017–1.600); *P* = 0.035]. In contrast, no statistically significant associations were observed between the other 14 micronutrients and ER+ breast cancer risk (*P* > 0.05 for all).

**Conclusion:**

Our results indicated that higher concentrations of vitamin B6 may be positively associated with ER+ breast cancer risk, and further research is needed to elucidate the underlying biological mechanisms of this association. This study provides new insights into understanding the role of micronutrients in breast cancer.

## 1 Introduction

According to the latest study published by the American Cancer Society, between 2012 and 2021, the incidence rate of breast cancer increased annually by 1%, particularly among women under 50 years of age. In 2024, it is anticipated that around 367220 new cases of breast cancer will be confirmed, with 42,250 women expected to die from breast cancer (McPherson et al., 2000). Approximately 70% of breast cancer cases are classified as hormone receptor-positive (HR+) malignancies, wherein tumor growth, survival, and progression are mechanistically driven by estrogen receptor (ER) expression and activation (Ríos-Hoyo et al., 2024). ER+ breast cancer is defined as ER-positive nuclear staining exceeding 1% in immunohistochemical (IHC) analysis (Hammond et al., 2010). Persistently elevated endogenous estrogen levels or prolonged exposure to exogenous estrogen may increase the risk of breast cancer (Wang et al., 2021).

Micronutrients primarily include vitamins and minerals, which are essential nutrients required to maintain human health. In recent decades, a growing body of research has underscored the potential role of micronutrient intake and supplementation in cancer prevention and risk reduction. A meta-analysis of prospective cohort study demonstrates an inverse association between elevated serum 25-hydroxyvitamin D concentration and reduced tumor incidence and mortality rates (Han et al., 2019). According to the findings of a randomized controlled trial (RCT), individuals with low serum vitamin B12 concentrations are at an elevated risk of developing non-cardia gastric adenocarcinoma (Miranti et al., 2017). Reduced serum selenium levels have been frequently observed in individuals diagnosed with prostate cancer (Oczkowski et al., 2021). Nevertheless, when it comes to micronutrient supplementation, more is not always better. Preclinical evidence from triple-negative breast cancer (TNBC) murine models reveals that supraphysiological vitamin B3 intake exacerbates tumor cell invasiveness and disrupts blood-brain barrier function, culminating in a marked elevation of cerebral metastatic risk (Maric et al., 2023). Vitamin B9 (also known as folate) plays an indispensable role in maintaining fundamental biological functions. However, a recent study has proposed an opposing perspective: Reducing folate intake in aged mice can effectively improve metabolic flexibility and help extend healthy lifespan (Blank et al., 2024).

**FIGURE 1:**
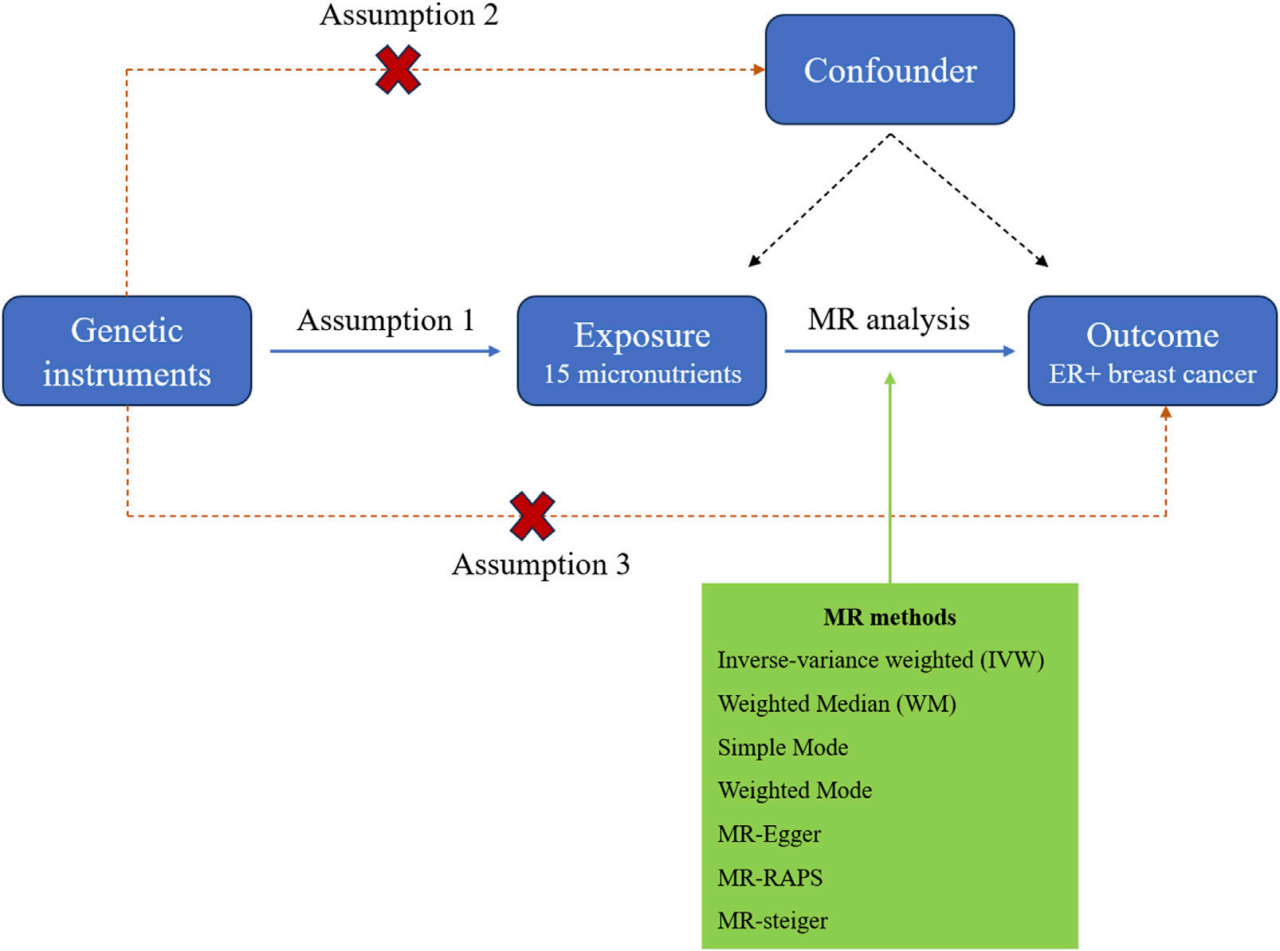
The study design diagram for MR analysis of the causal relationship between micronutrients and ER+ breast cancer risk.

Research on micronutrients and breast cancer risk is limited. Evidence primarily comes from observational studies using food frequency questionnaires (FFQs), which are less reliable and inherently prone to confounding and reverse causality. Besides, robust clinical trials assessing micronutrient effects are scarce (Zademohammadi et al., 2024; Key et al., 2011). Mendelian randomization (MR) is a genetic epidemiological approach that employs single-nucleotide polymorphisms (SNPs) as instrumental variables (IVs) to infer causal relationships between exposures and outcomes, utilizing publicly available summary statistics from large-scale genome-wide association studies (GWAS). As alleles are randomly allocated to gametes during meiosis, this effectively reduces the influence of confounding factors and reverse causality, thereby yielding more reliable research findings. Conducting an effective MR analysis requires meeting three key assumptions: 1) the genetic variants utilized in the study are strongly correlated with exposure; 2) genetic variants must not be influenced by factors that are associated with the selected exposure and outcome; and 3) genetic variants should affect the outcome only through the exposure (Emdin et al., 2017; Smith and Ebrahim, 2003). A conceptual diagram of the MR research framework is shown in Figure 1. Here, we conducted a two-sample MR analysis using seven methods to investigate the causal relationship between micronutrients and ER+ breast cancer, with micronutrients as the exposure factor and ER+ breast cancer as the outcome variable.

## 2 Materials and methods

### 2.1 GWAS data source for micronutrients

Genome-wide association study (GWAS) datasets for micronutrients were obtained from the MRC Integrative Epidemiology Unit (IEU) OpenGWAS project (https://gwas.mrcieu.ac.uk/; accessed December 2024) and the UK Biobank. Fifteen micronutrients were included: Copper (ieu-a-1073), Calcium (ukb-b-8951), Carotene (ukb-b-16202), Folate (ukb-b-11349), Iron (ukb-b-20447), Magnesium (ukb-b-7372), Potassium (ukb-b-17881), Selenium (ieu-a-1077), Vitamin A (ukb-b-9596), Vitamin B12 (ukb-b-19524), Vitamin B6 (ukb-b-7864), Vitamin C (ukb-b-19390), Vitamin D (ukb-b-18593), Vitamin E (ukb-b-6888), and Zinc (ieu-a-1079).

### 2.2 GWAS data source for ER+ breast cancer

We extracted publicly available data of ER+ breast cancer from FinnGen project (www.finngen.fi/en/), which included 14540 cases and 221,705 controls of European ancestry. There were no crossover samples between exposure and outcome GWAS datasets.

### 2.3 Selection of SNPs

SNPs associated with the 15 micronutrients were selected as IVs from GWASs. Genetic variants were confirmed to be independent at genome-wide significance (*P* < 5 × 10^−8^) with linkage disequilibrium (LD) r^2^ < 0.01. Weak instrument bias was assessed using F-statistics, with values ≥10 considered sufficient for MR analysis.

### 2.4 MR analysis

To evaluate the potential relationship between micronutrients and ER+ breast cancer, we applied seven different MR approaches: Inverse-variance weighted (IVW), Weighted Median (WM), Simple Mode, Weighted Mode, MR-RAPS (Robust adjusted profile score), MR-Steiger test and MR-Egger methods. The IVW method derives a comprehensive causal effect estimate by weighted averaging the causal effect estimates of each SNP. This approach takes into account the contribution of each SNP to the overall causal effect, ensuring minimal variance in the estimate and enhancing its accuracy. Therefore, The IVW method is regarded as the primary and most precise analytical approach in this study, with the other six methods as supporting methods. The odds ratio (OR) and 95% confidence interval (95% CI) were determined.

### 2.5 Sensitivity analysis

We conducted several sensitivity studies to evaluate the impact of pleiotropism on Mendelian random causality. Horizontal pleiotropy was detected by MR Egger test, while outliers in pleiotropy deviation were detected by MR-PRESSO (directional pleiotropy was absent if *P* > 0.05). Heterogeneity was evaluated through Cochran’s Q test, and when *P* > 0.05, SNP was considered to have no heterogeneity. Further, a “leave-one-out” analysis was performed to access the robustness of the data and whether any association was driven by any typical SNP.

### 2.6 Statistical analysis

All statistical analyses were performed using R software (v4.3.0) with the “TwoSampleMR” and “MRPRESSO” packages. A significance threshold of *P* < 0.05 was applied, and associations meeting this criterion were deemed statistically significant. Statistical power was also calculated via the formula approximation method in R software (v4.3.0). The Bonferroni correction was used to control false-positive results arising from multiple tests, and associations with *P* < 0.005 (0.05 divided by 10) were considered statistically significant.

## 3 Result

**FIGURE 2:**
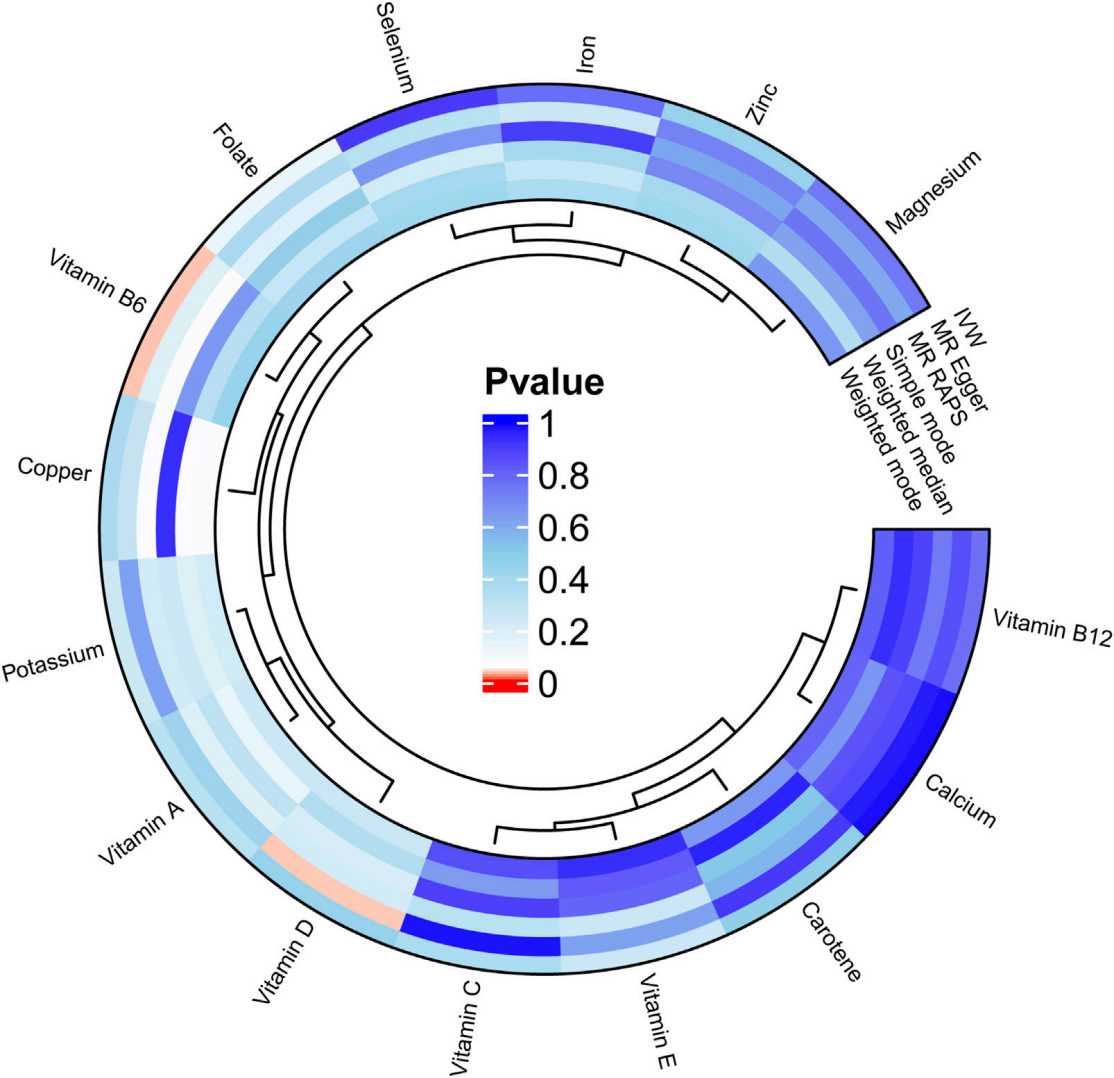
Circular diagram of MR analysis results for 15 micronutrients and ER+ breast cancer.

**FIGURE 3:**
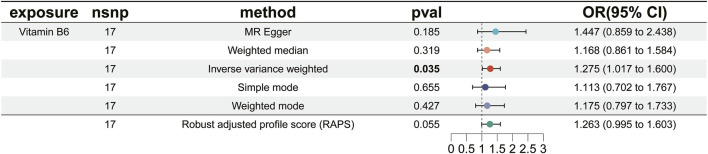
Forest plot of MR analysis results for vitamin B6 and ER+ breast cancer.

**FIGURE 4:**
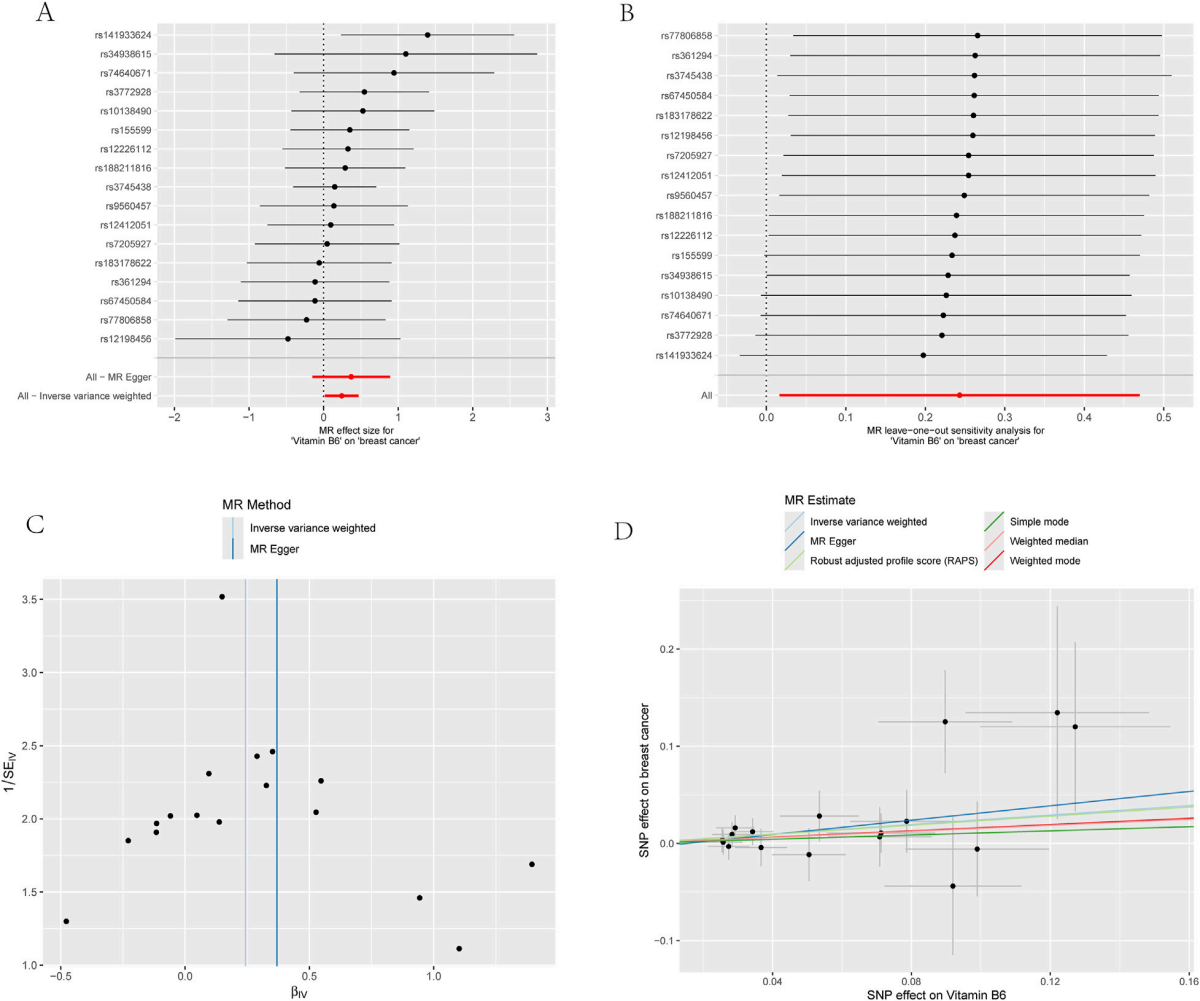
Four plots of all vitamin B6-related SNPs on the risk of ER+ breast cancer demonstrate that there is no horizontal pleiotropy. **(A)** Forest plot; **(B)** Leave-one-out plot; **(C)** Funnel plots; **(D)** Scatter plot.

We carried out this two-sample MR study to investigate the causal association between micronutrients and ER+ breast cancer. Figure 2 and Supplementary Table S1 illustrate six methods of MR analysis, and the results indicate that only vitamin B6, as genetically predicted, is associated with the incidence rate of ER+ breast cancer. The IVW results for vitamin B6 and ER+ breast cancer are [OR: 1.275; 95% CI: (1.017, 1.600); *P* = 0.035] (Table 1), and results of the other five methods suggest a consistent direction (Figures 3, 4D). Using the formula approximation method in R software, the statistical power for detecting the effect of vitamin B6 on ER+ breast cancer is calculated as 0.998, demonstrating sufficient power to detect an effect size of OR = 1.275. Meanwhile, the results of the MR-Steiger directional test indicate that there is no reverse causal relationship between vitamin B6 and ER+ breast cancer (*P* < 0.05) (Table 2). It can be considered that there may exist a potential causal relationship between vitamin B6 and ER+ breast cancer, and vitamin B6 may increase the risk of ER+ breast cancer. Genetics predicted that the rest 14 micronutrients had no impact on the development of ER+ breast cancer. However, after applying the Bonferroni adjusted significance level, the causal effect of vitamin B6 on ER+ breast cancer was no longer statistically significant (*P* = 0.530).

**TABLE 1:** MR analysis of the causal relationship between 15 micronutrients and ER+ breast cancer.

Exposure	Outcome	Sample size	nSNP	IVW OR (95%CI)	IVW *P*-value
Copper	ER (+) BC	1,073	6	0.969 (0.905–1.038)	0.374
Calcium	ER (+) BC	8,951	19	0.998 (0.800–1.245)	0.987
Carotene	ER (+) BC	16,202	15	0.916 (0.725–1.158)	0.463
Folate	ER (+) BC	11,349	12	1.240 (0.946–1.624)	0.119
Iron	ER (+) BC	20,447	11	1.043 (0.783–1.390)	0.773
Magnesium	ER (+) BC	7,372	17	0.940 (0.652–1.356)	0.741
Potassium	ER (+) BC	17,881	13	1.206 (0.880–1.651)	0.244
Selenium	ER (+) BC	1,077	6	0.995 (0.917–1.080)	0.907
Vitamin A	ER (+) BC	9,596	11	0.038 (6.053–23.626)	0.319
Vitamin B12	ER (+) BC	19,524	8	0.931 (0.584–1.483)	0.763
Vitamin B6	ER (+) BC	7,864	17	1.275 (1.017–1.600)	0.035
Vitamin C	ER (+) BC	19,390	10	0.869 (0.642–1.176)	0.363
Vitamin D	ER (+) BC	18,593	13	1.136 (0.818–1.577)	0.448
Vitamin E	ER (+) BC	6,888	12	0.850 (0.645–1.120)	0.248
Zinc	ER (+) BC	1,079	8	1.036 (0.946–1.135)	0.446

Abbreviations: SNP, single-nucleotide polymorphisms; IVW, inverse-variance weighted method; BC, breast cancer; OR, odds ratio; CI, confidence interval; ER, estrogen receptor.

**TABLE 2:** MR-Steiger test in this two-sample Mendelian randomization analysis.

Exposure	Outcome	SNP_r^2^.exposure	SNP_r^2^.outcome	Correct_causal _direction	Steiger_pval
Copper	ER (+) BC	0.080	9.23E-05	TRUE	6.15E-46
Selenium	ER (+) BC	0.064	4.71E-05	TRUE	2.44E-37
Vitamin B6	ER (+) BC	0.100	8.03E-05	TRUE	7.68E-59
Vitamin A	ER (+) BC	0.004	6.61E-05	TRUE	3.85E-37
Vitamin D	ER (+) BC	0.005	5.19E-05	TRUE	5.28E-49
Zinc	ER (+) BC	0.005	7.08E-05	TRUE	2.97E-43
Potassium	ER (+) BC	0.005	9.48E-05	TRUE	1.13E-40
Vitamin B12	ER (+) BC	0.004	5.06E-05	TRUE	1.99E-32
Calcium	ER (+) BC	0.003	7.61E-05	TRUE	1.42E-23
Vitamin E	ER (+) BC	0.004	4.35E-05	TRUE	6.51E-40
Magnesium	ER (+) BC	0.004	7.91E-05	TRUE	4.11E-38
Vitamin C	ER (+) BC	0.006	1.61E-04	TRUE	2.47E-48
Iron	ER (+) BC	0.006	6.74E-05	TRUE	2.58E-59
Carotene	ER (+) BC	0.007	7.69E-05	TRUE	2.85E-66
Folate	ER (+) BC	6.04E-04	6.64E-05	TRUE	8.48E-11

Abbreviations: SNP, single-nucleotide polymorphisms; BC, breast cancer; ER, estrogen receptor.

For the sensitivity analysis, both Cochran’s Q test and MR-PRESSO analysis for vitamin B6 and ER+ breast cancer association showed non-significant *P*-values (>0.05), suggesting no evidence of heterogeneity and horizontal pleiotropy (Table 3; Supplementary Table S2). Forest plot and funnel plot results of IVW and MR-Egger test showed that the distribution of SNP causal effect values was basically symmetrical, and no bias was found, therefore the results were relatively robust (Figures 4A,C). In the leave-one-out analysis, after eliminating SNPs one by one, the remaining SNPs were all located on the right side of the null line, yet the results remained unchanged, indicating the absence of SNPs significantly affecting the overall causality (Figure 4B).

**TABLE 3:** MR-PRESSO test result for directional pleiotropy.

Outcome	Exposure	RSSobs	MR-PRESSO *P* value
ER+ breast cancer	Vitamin B6	10.966	0.877

Abbreviations: MR, Mendelian randomization; ER, estrogen receptor.

## 4 Discussion

To evaluate the causative relationship between vitamin B6 and ER+ breast cancer, we carried out the two-sample MR analysis in this study. The results of our study implied that higher vitamin B6 intake may raise the risk of ER+ breast cancer.

Vitamin B6 is part of the B-vitamin family, which encompasses three forms: pyridoxine, pyridoxal, and pyridoxamine. Vitamin B6 is found in various sources, such as meat, fish, dairy products, and root vegetables (Barkoukis, 2016). In physiology, vitamin B6 has been proved to be involved in numerous biological processes: glycol-metabolism, lipid metabolism, amino acid synthesis, heme biosynthesis, and redox homeostasis, etc. Meanwhile, it occupies a central position in many disease mechanisms (Wang et al., 2024).

It has been shown in several studies that vitamin B6 is not only a tumor suppressor, but also a tumor promoter (Zuo et al., 2015). On the one hand, vitamin B6 exhibits antioxidant properties, modulates immune function, facilitates DNA repair, and regulates inflammatory responses, which collectively contribute to inhibiting cancer development (Stach et al., 2021). Oral consumption of a moderate dose of vitamin B6 has been shown to have a preventive effect on gastrointestinal cancer (Mocellin et al., 2017). A prospective cohort study reported that higher pre-diagnostic dietary vitamin B6 and choline intake levels improve the survival rate of ovarian cancer (Xu et al., 2022). On the other hand, Inappropriate vitamin B6 supplementation may inactivate key DNA repair enzymes, induce dysregulation of gene expression, and disrupt metabolic homeostasis, paradoxically promoting cancer progression (Calderon-Ospina et al., 2020). Chen CC’s work indicated that leukemic cells are addicted to the vitamin B6 pathway, and an epidemiological study showed that vitamin B6 is more likely to increase cancer risk instead of being a tumor protector in some solid tumors (Brasky et al., 2017; Chen et al., 2020). In a recently published retrospective study, researchers detected a high possibility of intrapulmonary metastasis in non-small cell lung cancer patients with high serum vitamin B6 levels after multivariable adjustment (Liu et al., 2023). As regard to breast cancer, the causal link between its development and vitamin B6 remains to be debatable. In the early 2000s, one population-based case-control study reported that breast cancer patients displayed higher serum vitamin B6 levels, while another study revealed that high vitamin B6 intake or serum levels is irrelevant to breast cancer risk (Franco et al., 2022). Literature also reported varying associations between vitamin B6 and the risk of different subtypes of breast cancer. No association between the ER, progesterone receptor (PR) and combined ER-PR status of breast cancers and vitamin B6 were reported in three cohort studies (Cho et al., 2007; Maruti et al., 2009; Shrubsole et al., 2011). However, one prospective study with a mean follow-up of 16.3 years presented that high vitamin B6 was associated with reduced risk of ER+ breast cancer and human epidermal growth factor receptor 2 negative (HER2-) breast cancer (Cancarini et al., 2015). Conclusion from a prospective study in 2019 demonstrated that in summary, folate, vitamin B12, vitamin B6, homocysteine, and cysteine levels are independently associated with breast cancer risk, regardless of *in situ*/invasive, hormone receptor status, or tumor molecular subtype (Houghton et al., 2019). A recently published MR study found that vitamin B6 was associated with a higher risk of triple-negative breast cancer (OR = 1.361, 95%CI = 1.04–1.78, *P* = 0.0248) but not with the other breast cancer subtypes (Kim et al., 2023). Owing to methodological constraints of traditional research designs, neither potential reverse causation nor heterogeneity can be entirely excluded. In our comprehensive MR analysis, the result supported an unexpected positive association between the elevated levels of vitamin B6 and the risk of ER+ breast cancer. Vitamin B6 showed marginal significance in the unadjusted analysis (*P* = 0.035), and it failed to retain significance following Bonferroni correction (adjusted *P* = 0.530). Given the multiple comparisons across 15 tests, this finding may represent a false-positive signal. Nevertheless, in light of its potential biological plausibility (Brasky et al., 2017; Chen et al., 2020; Liu et al., 2023), the association warrants further investigation and validation in larger cohorts. Since the mechanisms by which vitamin B6 promotes ER+ breast cancer risk remain incompletely understood, and the relevant literature is limited, we primarily propose potential hypothetical mechanisms by integrating vitamin B6’s biological properties with relevant pathways in ER+ breast cancer cells. The possible mechanisms may be as follows. Firstly, vitamin B6 modulates cellular proliferation in cancer cells by facilitating amino acid metabolism and nucleotide synthesis. It additionally mediates apoptosis through the regulation of oxidative stress, redox homeostasis, and epigenetic modifications. An excess of vitamin B6 could promote tumor growth by modulating the redox-mediated metabolic pathways, as solid tumors critically depend on high dynamic amino acid turnover to sustain their survival and growth (Parra et al., 2018; Frost et al., 2025). Secondly, one-carbon metabolism is an essential pathway in organisms, which is linked to DNA synthesis, methylation, repair and amino acid metabolism (Petrova et al., 2023). Vitamin B6, one of the one-carbon metabolism-related vitamin, is capable of playing a role in the progression of tumors through influencing the stability of DNA and activating antioxidant enzymes (Selhub et al., 2013; Song et al., 2022). De Vogel et al. conducted the Netherlands Cohort Study on diet and cancer (n = 120,852) and found that high vitamin B6 intake has a positive correlation with the development of tumors showing mutL homologue 1 (MLH1) hypermethylation (de Vogel et al., 2008). Compared with healthy individuals, a significantly higher frequency of MLH1 hypermethylation is observed in breast cancer patients (Nikitin et al., 2020). In the meantime, dysregulation of the MutL mismatch repair complex (MLH1/3, PMS1/2), including MLH1 promoter hypermethylation, is linked to endocrine therapy and chemotherapy resistance in ER+ breast cancer (Haricharan et al., 2017; Dasgupta et al., 2019), suggesting that MLH1 hypermethylation may correlate with poor prognosis of ER+ breast cancer. Building upon existing epidemiological and experimental evidence, we hypothesize that excessive vitamin B6 may increase ER+ breast cancer risk by promoting MLH1 hypermethylation. Thirdly, vitamin B6 may promote ER+ breast cancer risk by modulating inflammatory signaling pathways. Previous data have suggested that vitamin B6 modulate inflammatory pathways via cofactor roles in homocysteine and tryptophan/kynurenine metabolism (Ueland et al., 2017). Abnormalities in the tryptophan/kynurenine metabolism are potentially linked to the occurrence, progression, and treatment response of breast cancer. Indoleamine 2,3-dioxygenase 1 (IDO1) is a key rate-limiting enzyme in the kynurenine pathway (Ding et al., 2024). Vitamin B6 facilitates the enzymatic activity of IDO1 through the provision of pyridoxal 5-phosphate (PLP)as a cofactor (Majewski et al., 2016; Li et al., 2019; Huang et al., 2022). In ER+ breast cancer, increased IDO1 expression may deplete tryptophan, suppress T cells, and aid immune evasion. Besides, ER signaling might activate IDO1, creating an “ER-IDO1 immune suppression axis” that strengthens tumor resistance to immunotherapy (Soliman et al., 2013; Fumagalli et al., 2016; Anurag et al., 2020). These three mechanistic hypotheses we propose collectively suggest that excessive vitamin B6 intake may promote ER+ breast carcinogenesis.

Our study exhibits several methodological strengths. First, the MR analysis adheres to the principle of genetic randomization, substantially reducing confounding bias. Second, the utilization of large sample size summary data provides strong evidence compared to conventional observational studies. Additionally, we implemented multiple sensitivity analyses to ensure the robustness of the causal inference.

Nevertheless, this study has several notable limitations: (1) Both exposure and outcome data were derived exclusively from European populations. The generalizability of findings to other ethnic groups may be constrained due to unaccounted population stratification effects; (2) Stratified analyses of potentially relevant clinical variables (e.g., menopausal status, body mass index) were precluded by insufficient clinical data; (3) While our two-sample MR analysis suggests a potential causal link between vitamin B6 and ER+ breast cancer with borderline statistical significance, large-scale clinical studies with mechanistic investigations are required to validate these findings and clarify the underlying biological pathways.

## 5 Conclusion

We employed this two-sample MR analysis to genetically investigate the causal relationship between vitamin B6 and ER+ breast cancer. Our findings suggest a potentially significant causal association, indicating that elevated vitamin B6 levels may increase the risk of ER+ breast cancer. This study highlights that while vitamin B6 supplementation may confer health benefits in certain contexts, excessive or prolonged use could pose potential health risks. Specifically, individuals at high risk of breast cancer should avoid indiscriminate vitamin B6 supplementation.

## Data Availability

The datasets presented in this study can be found in online repositories. The names of the repository/repositories and accession number(s) can be found in the article/Supplementary Material.

## Abbreviations

MR, mendelian randomization; ER+, estrogen receptor-positive; BC, breast cancer; IV, instrumental variable; GWAS, genome-wide association study; OR, odds ratio; CI, confidence interval; SNP, single nucleotide polymorphism; HR+, hormone receptor-positive; IHC, immunohistochemical; RCT, randomized controlled trial; TNBC, triple-negative breast cancer; FFQ, food frequency questionnairy; IEU, Integrative Epidemiology Unit; IVW, Inverse-variance weighted; WM, Weighted Median; RAPS, Robust adjusted profile score; LD, linkage disequilibrium; PR, progesterone receptor; HER2-, human epidermal growth factor receptor 2 negative; MLH1, mutL homologue 1; PLP, pyridoxal 5-phosphate; IDO1, Indoleamine 2,3-dioxygenase 1.
