# Supplementary material for: Genetic prediction of the casual relationship between micronutrients and ER+ breast cancer: a Mendelian randomized study
Source: Front Genet. 2025 Jul 23;16:1599724. doi: 10.3389/fgene.2025.1599724 (PMC12325068; doi:10.3389/fgene.2025.1599724)
Supplement: Supplementary file 1 [file Table1.docx]

**Supplementary** **Table S1. Results of MR analysis studying the effect of** **15** **micronutrients on ER+ breast cancer.**

| **Micronutrients** | **Methods** | **OR** | **95%CI** | ***P*** |
| --- | --- | --- | --- | --- |
| Copper | MR-egger | 0.940 | (0.853-1.034) | 0.280 |
|  | Weight median | 0.952 | (0.903-1.003) | 0.063 |
|  | IVW | 0.969 | (0.905-1.038) | 0.374 |
|  | Simple mode | 1.004 | (0.904-1.115) | 0.942 |
|  | Weighted mode  MR-RAPS | 0.939  0.952 | (0.895-0.985)  (0.904-1.003) | 0.049  0.064 |
| Selenium | MR-egger | 0.908 | (0.769-1.072) | 0.318 |
|  | Weight median | 0.968 | (0.898-1.044) | 0.398 |
|  | IVW | 0.995 | (0.917-1.080) | 0.907 |
|  | Simple mode | 1.108 | (0.953-1.290) | 0.241 |
|  | Weighted mode  MR-RAPS | 0.961  0.982 | (0.882-1.048)  0.906-1.064 | 0.411  0.651 |
| Zinc | MR-egger | 0.936 | (0.671-1.306) | 0.712 |
|  | Weight median | 1.039 | (0.951-1.134) | 0.399 |
|  | IVW | 1.036 | (0.946-1.135) | 0.446 |
|  | Simple mode | 0.963 | (0.808-1.149) | 0.689 |
|  | Weighted mode  MR-RAPS | 1.050  1.026 | (0.928-1.189)  (0.930-1.131) | 0.462  0.612 |
| Folate | MR-egger | 1.345 | (0.728-2.486) | 0.367 |
|  | Weight median | 1.239 | (0.873-1.759) | 0.230 |
|  | IVW | 1.240 | (0.946-1.624) | 0.119 |
|  | Simple mode | 1.286 | (0.652-2.538) | 0.483 |
|  | Weighted mode  MR-RAPS | 1.301  1.213 | (0.696-2.432)  (0.922-1.595) | 0.427  0.168 |
| Carotene | MR-egger | 1.029 | (0.619-1.711) | 0.914 |
|  | Weight median | 1.009 | (0.724-1.404) | 0.959 |
|  | IVW | 0.916 | (0.725-1.158) | 0.463 |
|  | Simple mode | 1.226 | (0.696-2.159) | 0.492 |
|  | Weighted mode  MR-RAPS | 1.123  0.926 | (0.696-1.812)  (0.715-1.200) | 0.643  0.563 |
| Potassium | MR-egger | 0.801 | (0.337-1.907) | 0.627 |
|  | Weight median | 1.321 | (0.893-1.952) | 0.163 |
|  | IVW | 1.206 | (0.880-1.651) | 0.244 |
|  | Simple mode | 1.538 | (0.774-3.057) | 0.243 |
|  | Weighted mode  MR-RAPS | 1.469  1.217 | (0.814-2.651)  (0.911-1.625) | 0.226  0.183 |
| Vitamin D | MR-egger | 3.520 | (1.253-9.891) | 0.036 |
|  | Weight median | 1.218 | (0.827-1.794) | 0.319 |
|  | IVW | 1.136 | (0.818-1.577) | 0.448 |
|  | Simple mode | 1.834 | (0.723-4.654) | 0.226 |
|  | Weighted mode  MR-RAPS | 1.822  1.214 | (0.679-4.889)  (0.845-1.746) | 0.257  0.295 |
| Vitamin C | MR-egger | 1.009 | (0.441-2.309) | 0.983 |
|  | Weight median | 0.911 | (0.618-1.342) | 0.637 |
|  | IVW | 0.869 | (0.642-1.176) | 0.363 |
|  | Simple mode | 0.954 | (0.465-1.955) | 0.901 |
|  | Weighted mode  MR-RAPS | 1.050  0.855 | (0.606-1.819)  (0.632-1.157) | 0.867  0.310 |
| Vitamin B12 | MR-egger | 0.894 | (0.270-2.960) | 0.860 |
|  | Weight median | 1.017 | (0.667-1.550) | 0.939 |
|  | IVW | 0.931 | (0.584-1.483) | 0.763 |
|  | Simple mode | 1.047 | (0.559-1.958) | 0.891 |
|  | Weighted mode  MR-RAPS | 1.064  0.929 | (0.631-1.794)  (0.600-1.438) | 0.822  0.742 |
|  | MR-egger | 0.597 | (0.260-1.374) | 0.256 |
|  | Weight median | 0.794 | (0.537-1.174) | 0.247 |
| Iron | IVW | 1.043 | (0.783-1.390) | 0.773 |
|  | Simple mode | 0.711 | (0.365-1.385) | 0.340 |
|  | Weighted mode  MR-RAPS | 0.727  1.033 | (0.375-1.411)  (0.770-1.386) | 0.368  0.829 |
| Vitamin E | MR-egger  Weight median  IVW  Simple mode  Weighted mode  MR-RAPS | 1.155  1.036  0.850  0.930  1.015  0.869 | (0.666-2.000)  (0.746-1.440)  (0.645-1.120)  (0.528-1.638)  (0.694-1.486)  (0.683-1.106) | 0.619  0.831  0.248  0.805  0.939  0.253 |
| Magnesium | MR-egger  Weight median  IVW  Simple mode  Weighted mode  MR-RAPS | 1.218  0.837  0.940  0.838  0.864  0.948 | (0.591-2.513)  (0.581-1.204)  (0.652-1.356)  (0.559-1.958)  (0.419-1.676)  (0.682-1.319) | 0.601  0.338  0.741  0.625  0.623  0.752 |
| Vitamin B6 | MR-egger  Weight median  IVW  Simple mode  Weighted mode  MR-RAPS | 1.447  1.168  1.275  1.113  1.175  1.263 | (0.859-2.434)  (0.856-1.594)  (1.017-1.600)  (0.701-1.769)  (0.788-1.754)  (0.995-1.603) | 0.185  0.328  0.035  0.656  0.441  0.055 |
|  | MR-egger | 0.985 | (0.422-2.302) | 0.973 |
|  | Weight median | 1.070 | (0.805-1.421) | 0.643 |
| Calcium | IVW | 0.998 | (0.800-1.245) | 0.987 |
|  | Simple mode | 0.948 | (0.555-1.618) | 0.846 |
|  | Weighted mode  MR-RAPS | 1.065  0.980 | (0.682-1.664)  (0.780-1.232) | 0.785  0.864 |
| Vitamin A | MR-egger  Weight median  IVW  Simple mode  Weighted mode  MR-RAPS | 2.97E-05  0.002  0.038  0.0003  0.0002  0.036 | (6.61E-16-1333755)  (7.71E-07-6.768)  (6.05E-05-23.626)  (1.87E-10-573.066)  (1.65E-10-172.604)  (0-9.961) | 0.426  0.136  0.319  0.300  0.247  0.247 |

Abbreviations: MR, Mendelian randomization; IVW, Inverse variance weighted; ER+, Estrogen receptor-positive; RAPS, Robust adjusted profile score

| **Supplementary Table S2. Heterogeneity and pleiotropy analysis of causal relationships between 15 micronutrients on ER+ breast cancer.** | | | | | | | | | |
| --- | --- | --- | --- | --- | --- | --- | --- | --- | --- |
| **Outcome** | **Exposure** | **nSNP** | **Heterogeneity analysis** | | | | **Pleiotropy analysis** | | |
|  |  |  | **IVW** | | **MR-Egger** | | **MR-Egger** | | |
|  |  |  | **Q** | **Q_pval** | **Q** | **Q_pval** | **Intercept** | **Se** | ***P* value** |
| ER+ breast cancer | Copper | 6 | 18.829 | 0.002 | 15.685 | 0.003 | 0.015 | 0.017 | 0.421 |
|  | Selenium | 6 | 11.096 | 0.049 | 8.065 | 0.089 | 0.022 | 0.018 | 0.287 |
|  | Zinc | 8 | 17.527 | 0.014 | 16.463 | 0.011 | 0.021 | 0.033 | 0.556 |
|  | Folate | 12 | 12.782 | 0.308 | 12.673 | 0.243 | -0.004 | 0.013 | 0.776 |
|  | Carotene | 15 | 11.734 | 0.628 | 11.478 | 0.571 | -0.005 | 0.011 | 0.621 |
|  | Potassium | 13 | 14.956 | 0.244 | 13.732 | 0.248 | 0.016 | 0.016 | 0.343 |
|  | Vitamin D | 13 | 20.151 | 0.064 | 13.867 | 0.240 | -0.049 | 0.022 | 0.047 |
|  | Vitamin C | 10 | 10.941 | 0.280 | 10.743 | 0.217 | -0.006 | 0.016 | 0.711 |
|  | Vitamin B12 | 8 | 17.738 | 0.013 | 17.722 | 0.007 | 0.002 | 0.027 | 0.944 |
|  | Iron | 11 | 10.167 | 0.119 | 8.220 | 0.512 | 0.020 | 0.014 | 0.196 |
|  | Vitamin E | 12 | 16.652 | 0.6518 | 14.396 | 0.156 | -0.016 | 0.013 | 0.239 |
|  | Magnesium | 17 | 37.790 | 0.002 | 36.811 | 0.002 | -0.013 | 0.015 | 0.427 |
|  | Vitamin B6  Calcium  Vitamin A | 17  19  11 | 10.078  12.638  14.202 | 0.862  0.813  0.164 | 9.800  12.637  13.669 | 0.832  0.760  0.135 | -0.006  0.0004  0.014 | 0.011  0.015  0.024 | 0.605  0.975  0.567 |

Abbreviations: MR, Mendelian randomization; IVW, Inverse variance weighted; ER+, Estrogen receptor-positive
